# Allogeneic uterus transplantation in a rhesus model: A short-term graft viability study

**DOI:** 10.1371/journal.pone.0243140

**Published:** 2020-12-17

**Authors:** Bo Yu, Zhongyu Liu, Chunyu Zhang, Yu Wu, Jinsong Han, Huajun Li, Bing Xu, Hongyan Guo, Kun Zhang

**Affiliations:** Department of Gynecology and Obstetrics, Peking University Third Hospital, Beijing, PR China; University of Insubria, ITALY

## Abstract

**Objective:**

This study examined the feasibility and safety of allogeneic uterus transplantation (UTx) from a living donor and assessed short-term graft viability in a rhesus model. This research is an important step towards further clinical application of UTx in humans.

**Materials and methods:**

Four female rhesus monkeys with regular menstrual cycles were used in this study, the animals were either donors or recipients depending on ABO blood type compatibility. Retrieval surgery was performed to connect the uterus and uterine arteries together with the ovarian uterine vein from the living donor, and the vagina of the recipient was excised. After the back table had been prepared, bilateral uterine arteries were anastomosed end-to-side with the iliac externa arteries, and bilateral ovarian uterine veins were anastomosed end-to-side with the iliac externa vena. The transplanted uterus was evaluated based on the conditions of arterial blood, and flow was evaluated by transabdominal ultrasonography one month post operation. The conditions of the transplanted uterus were examined by secondary laparotomy. The reproductive function of rhesus monkeys was evaluated on the basis of the menstrual cycle.

**Results:**

All 4 rhesus monkeys received the transplantation surgery without any surgical complications. No injury occurred in the other organs, and no vascular injury was observed in the allogeneic uterus. All recipients survived after the surgery with a 100% short-term survival rate. All recipients resumed normal menstruation within two months after surgery.

**Conclusions:**

Our short follow-up shows that allogeneic UTx surgery is a safe and feasible technology in the rhesus model. The arterial conditions and blood flow of the grafted uterus can be monitored by ultrasonography examination.

## Introduction

Uterine factor infertility (UFI) is a major type of female infertility that is still considered untreatable. Available options include surrogacy and adoption. However, surrogacy is illegal in many nations including China [1–3]. On the other hand, due to the traditional culture in China, adoption is not as common as in it is in Western countries. Uterus transplantation (UTx) is a potential option for women with UFI who wish to have a child. Accumulative data from previous experimental studies have led to the clinical application of UTx. In 2014, almost 50 years after the initial concept was first proposed, the first live birth following UTx was reported [4]. The investigators suggest that UTx may be a feasible fertility-restoring intervention for women with UFI. Thus far, more than 60 UTx surgeries have been performed globally, and 18 offspring have been reported [5].

In recent decades, organ transplantation has been used in clinical practice. At the same time, assisted reproductive technology (ART) has progressed rapidly for live births after in vitro fertilization (IVF). The technology of human UTx benefits from the development of those technologies. Many clinical study teams have recently aimed for the clinical application of UTx on the basis of experimental data. However, many clinical and technical issues of UTx need to be resolved [6]. Since the parameters associated with UTx have not been standardized, basic data from animal experiments are needed for the further clinical application of UTx.

Animal models are extensively used for the investigation of human disease and surgical procedures, such as endometriosis and organ transplantation. In recent years, animal studies on UTx have been performed with rats, rabbits, mice, dogs, swine, sheep, and nonhuman primates [7]. The outcomes of allogeneic UTx have been reported only in rats and sheep [8, 9], and there are only a few cases of UTx in nonhuman primates [10, 11]. UTx cases involving nonhuman primates would provide important preclinical guidance to resolve problems associated with UTx in clinical practice, since these animals are anatomically and physiologically similar to humans.

The potential challenges of allogeneic UTx in nonhuman primates pertain to complicated surgical techniques, potential ischemia-reperfusion injury and rejection, and the delicate techniques used for vascular anastomosis. The evaluation criteria for the success of UTx include long-term survival of recipient animals and recovery of reproductive function. The anatomical pelvic structures of rhesus monkeys are almost identical to those of humans, and the experience of allogenic UTx in nonhuman primates can be used to investigate transplantation in humans. In this context, we aimed to evaluate the surgical technique for allogeneic UTx in a rhesus model.

## Materials and methods

### Animals

Four female rhesus monkeys with regular menstrual cycles were purchased from an animal experimental center. The mean body weight of all the rhesus monkeys was 5.325 kg, while no pregnancy was noted preoperatively. To reduce postoperative immune rejection, the animals were selected as either donors or recipients (n = 4) on the basis of ABO blood type compatibility. The general condition of each rhesus monkey was examined 1–2 weeks before surgery. After the experimental animal was verified to be in good condition post surgery, she was housed for use in further research, such as embryo transplantation.

All the rhesus monkeys were acclimatized to their new surroundings for at least 7 days to reduce possible pressure. All rhesus monkeys were housed in separate cages with adequate food and water. The room temperature varied from 19°C to 25°C, with a relative humidity of 40–60% and a 12-hrs light/dark cycle.

Animal health and behavior were monitored twice daily. The animal was euthanized within 4 hrs if any of the following conditions occurred: weight loss, pain that could not be effectively controlled, excessive tumor growth or ascites production, persistent self-harm behavior, systemic hair loss caused by disease, long-term diarrhea that could not be treated due to experimental factors, persistent fatigue accompanied by rough fur, an arched back, an enlarged abdomen, inability to walk, severe anemia, jaundice, abnormal central nervous system response, uncontrollable bleeding, abnormal urination, disorders affecting eating and drinking, end-stage infectious diseases, severe low temperature, and obvious functional impairment. In this study, no animals died before meeting the criteria for euthanasia.

All personnel participating in the animal experiment received animal experiment training and obtained the qualification certificate of animal experiment practitioners. The study was performed in accordance with the recommendations in the Guide for the Care and Use of Laboratory Animals of the National Research Council and approved by the Ethics Committee of Peking University Third Hospital (No. LA2016317).

### Blood group compatibility test

The blood group compatibility test method included bloodgrouping and crossmatching. The reagents were added in the following order: 1) human anti-A erythrocyte suspension + serum to be tested; 2) human anti-B erythrocyte suspension + serum to be tested; 3) human anti-B serum + erythrocyte suspension to be measured; and 4) human anti-A serum + erythrocyte suspension to be measured. The major and minor crossmatch tests were included in the compatibility test [12]. The major crossmatch tests for alloantibodies in the recipient’s plasma against donor red cells and the minor crossmatch tests for alloantibodies in the donor’s plasma against the recipient’s red blood cells. The presence of agglutination on either test indicated that the recipient was not compatible with the donor’s red blood cells (major) or with the donor’s plasma (minor) [13].

### Anesthesia protocol

The hair of each rhesus monkey was clipped on the lower abdominal region, and food was withdrawn 24 hrs before the day of surgery. After being sedated, the animal was injected with 3% pentobarbital sodium (Shanghai Harring Biotechnology Co., Ltd). The abdomen was sterilized, the animal was placed in the supine position, and vein puncture catheterization was performed. A tracheal tube was inserted with a positive airway pressure breathing machine connected, and anesthesia was maintained with 3% pentobarbital sodium (Shanghai haring Biotechnology Co., Ltd.). All animals received continuous intravenous infusion to compensate for the loss of fluid during surgery. For prophylaxis antibiotics were administered from the initiation of the operation until one week after surgery.

### Uterus transplantation

**Fig 1:**
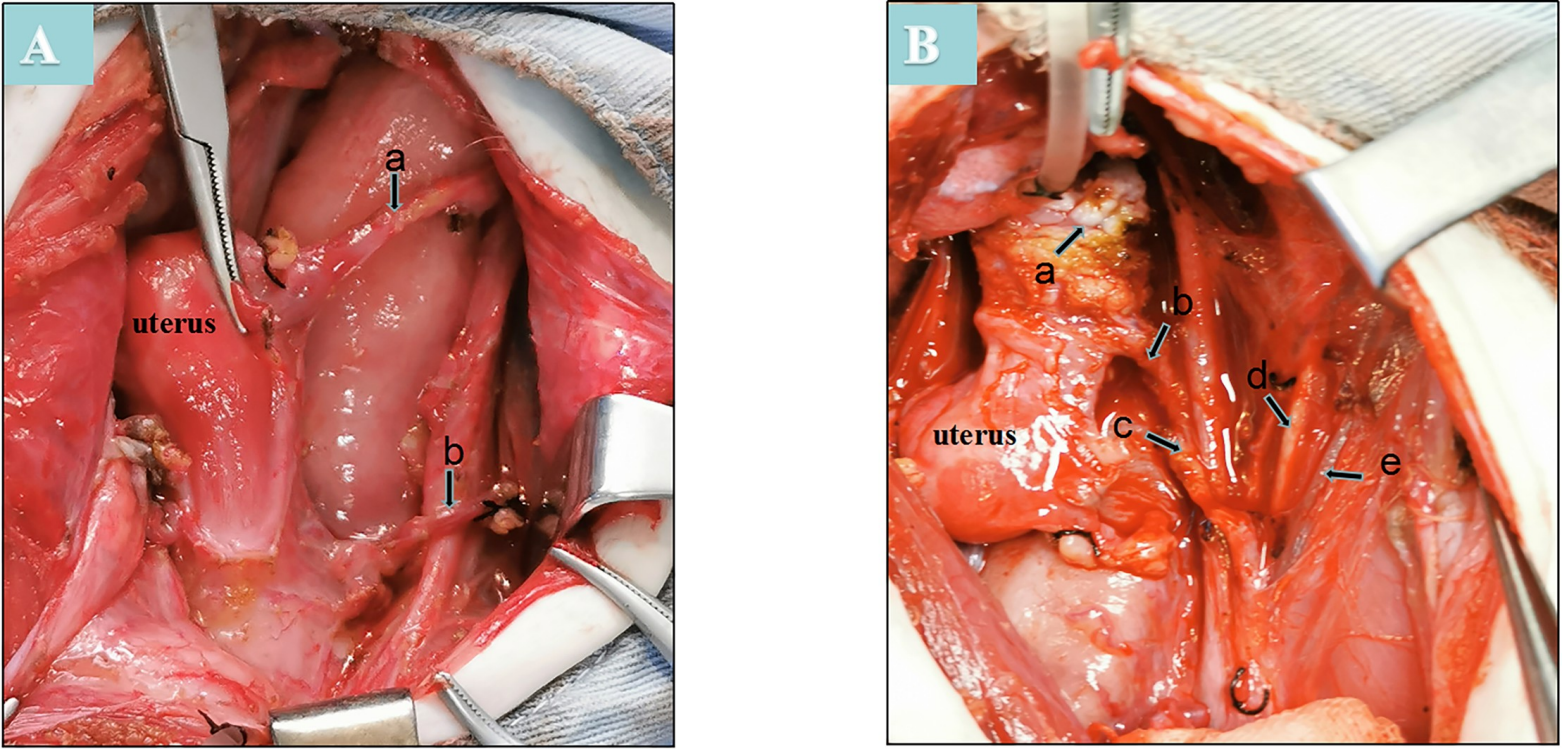
An overview of the anatomical reproduction and iliac vessels in the rhesus model. A: a-ovarian uterine veins; b-uterine artery. B: a-cervical; b-uterine artery; c-internal iliac artery; d-external iliac artery; e-external iliac veins.

**Fig 2:**
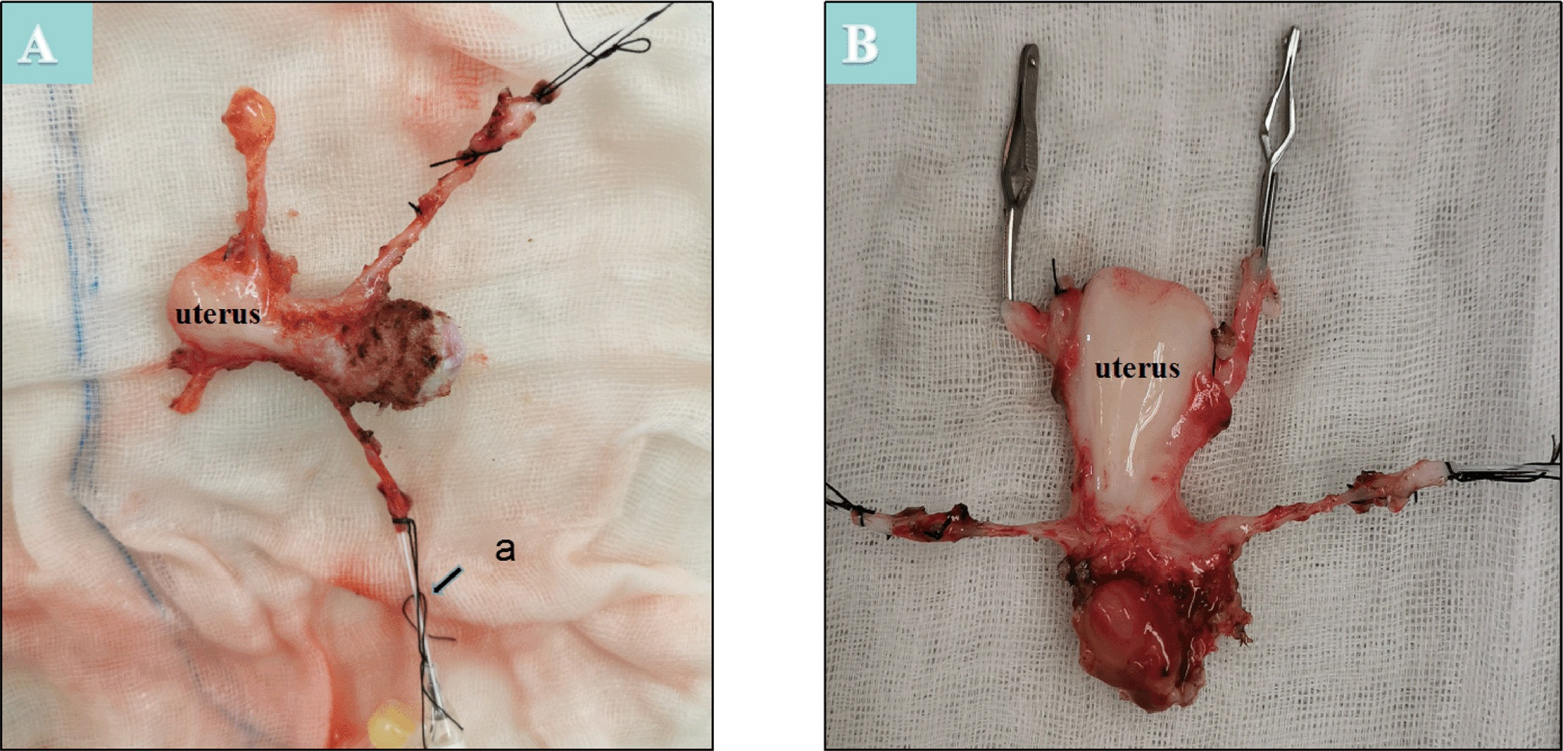
The isolated uterine tissue was stored at 4°C using organ preservation solutions. A: a-cold histidine-tryptophan-ketoglutarate solution via a perfusion catheter (22-G intravenous needle)-perfused uterine artery. B: Lavage outflow clear and pale uterus.

**Fig 3:**
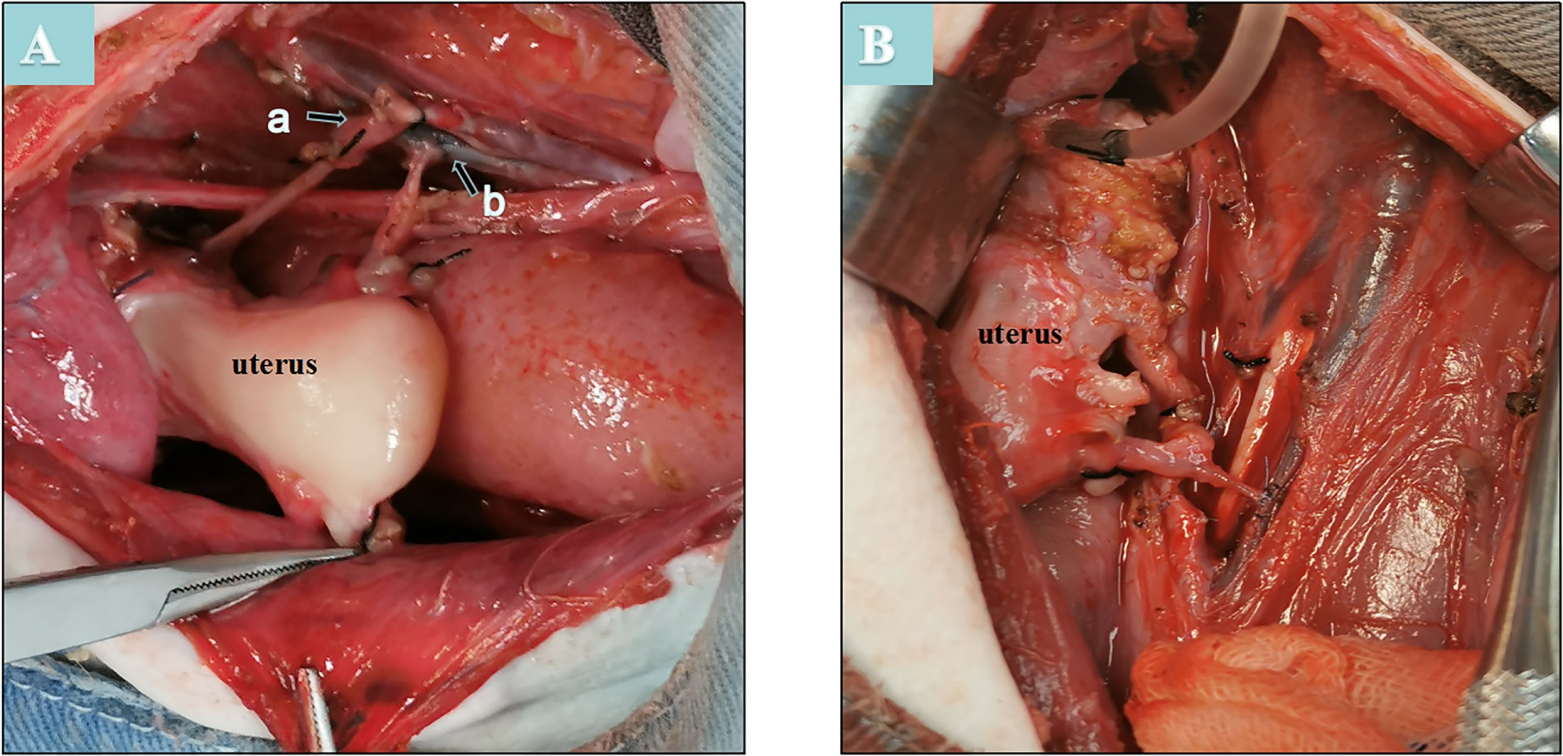
A: a-Transplantation of the internal iliac artery end-to-side anastomosis with the external iliac artery from the recipient, b-Transplantation of the ovarian uterine veins end-to-side anastomosis with the external iliac veins from the recipient. B: The grafting rapid vascular filling after anastomosis.

First, laparotomy through a midline incision was performed in the donor animal. The uterine artery, adjacent internal iliac artery, and ovarian uterine veins were dissected separately (Fig 1A). Then, the vaginal artery and vessels surrounding the uterus were dissected separately (Fig 1B). The ovaries and oviducts of donors were preserved in situ (Fig 1A). The isolated uterine tissue was stored at 4°C in organ preservation solutions and perfused with 300–350 milliliters (ml) of cold histidine-tryptophan-ketoglutarate solution (HTK®; Custodiol®, Nordmedica, Gentofte, Denmark) via a perfusion catheter (22-G intravenous needle) in the uterine artery (Fig 2A). The uterus was maintained under these conditions until the lavage outflow became clear (Fig 2B). During recipient surgery, the uterine graft was brought into the operative field, and the vagina of the graft was anastomosed with the vaginal vault of the recipient to fix the uterus in the pelvis (Fig 3A). Before vascular anastomoses, heparin (100 IU) was administered intravenously. The vascular clamps were placed on both ends of the isolated segments of the external iliac artery. Then, the bilateral internal iliac artery was anastomosed end-to-side with the iliac externa of the arteries, and bilateral ovarian uterine veins were anastomosed end-to-side with the vena iliac externa using continuous sutures (7–0 or 8–0 Prolene®) (Fig 3B). The clamps were released after the conclusion of all vascular anastomoses, and homeostasis was evaluated. The severe leakage was controlled by placing additional sutures. Anastomotic patency and blood flow were assessed by the pulsations of the uterine arteries and the color of the uterus. The vaginal cuff was then reattached to the vagina by interrupted sutures (1–0 polyglactin).

### Postoperative management

**Fig 4:**
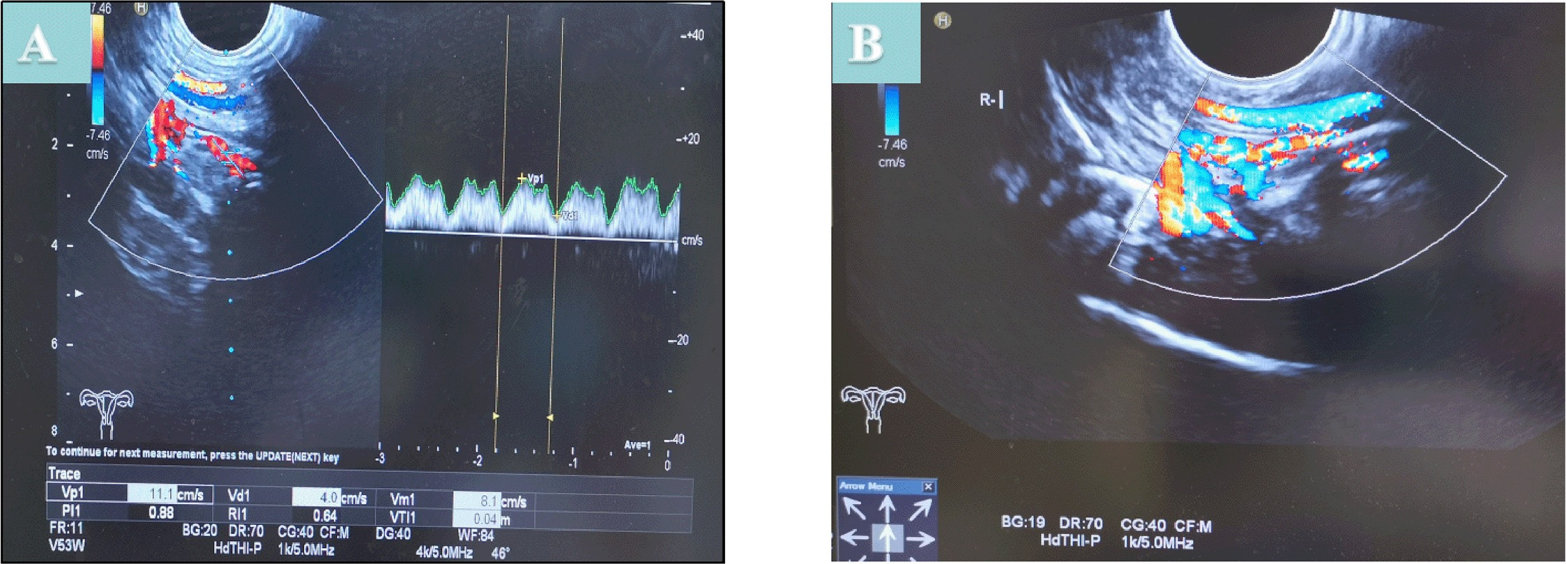
The blood flow of the transplanted uterus was evaluated by transabdominal ultrasonography.

The size and blood flow of the transplanted uterus were determined by transabdominal ultrasonography after closing the abdominal incision by interrupted sutures (1–0 polyglactin) (Fig 4). After the surgical procedure, animals were separately accommodated in a single cage. The general condition of each rhesus monkey in terms of the following aspects was evaluated daily: appetite, bowel movements, vomiting, urination, attitude, and laparotomy wound site. To monitor for potential rejection after surgery, the size of the transplanted uterus and blood flow in the transplanted uterine artery were determined by transabdominal ultrasonography under anesthesia in the first and fourth weeks after the operation. Cervical tissue biopsy was performed before the operation, 5 days after the operation, and 25 days after the operation. Dexamethasone (DXM) was administered to all animals as an induction treatment. Mycophenolate mofetil (MMF), cyclosporine a (CyA), and tacrolimus were administered orally.

## Results

### Surgical parameters

In total, four rhesus monkeys were used in this study. The animals were designated as either a donor or recipient depending on their ABO blood type compatibility. The details of the UTx operation parameters of each animal are summarized in Table 1. The average time of donor surgery was 235 minutes (min); the average time of uterine retrieval was 133 min; the average time of warm ischemia 1^*^ was 9 minutes; the average time of warm ischemia 2^*^ was 101.75 min; the average time of cold ischemia was 86.5 min; the average perfusion time was 23.75 min; the average vascular anastomosis time was 133 min; and the average blood loss was 20 milliliters (ml).

**Table 1:** Uterus transplantation surgery parameters in 4 rhesus monkeys.

NO.	Donor surgery	Retrieval	Warm ischemia (min)		Cold ischemia*	Perfusion	Vascular anastomosis	Hemorrhage
	(min)	(min)	1*	2*	(min)	(min)	(min)	(ml)
1	260	152	10	113	131	30	152	20
2	250	150	11	123	120	30	160	30
3	210	115	7	82	35	15	115	15
4	220	115	8	89	60	20	115	15

*: warm ischemia 1 (donor): ischemia during implantation, from removal of the organ from ice until reperfusion; warm ischemia 2 (recipient): from cold storage to transplant vascular anastomosis completion and re-warming; cold ischemia: from organ retrieval to before vascular anastomosis.

### Immunosuppression

Dexamethasone (5 mg/day) was orally administered to all animals on the day before surgery as an induction treatment until two weeks post operation (Table 2). MMF was administered orally 250 mg/day post operation, CyA was administered orally 25 mg/day post operation, and tacrolimus was administered orally 1 mg/day. No rejection reaction occurred in any rhesus monkey.

**Table 2:** Immunosuppressive therapy.

Drug	Method	Dose
DXM	orally	5 mg/day
MMF	orally	250 mg/day
CyA	orally	25 mg/day
Tacrolimus	orally	1 mg/day

DXM: dexamethasone; MMF: mycophenolate mofetil; CyA: cyclosporine a.

### Assessment of graft viability

The short-term outcome evaluation showed that the transplanted vessels had satisfactory intraoperative conditions in all rhesus monkeys. Doppler ultrasonography revealed that the blood flow of the external iliac artery was stable in all four animals at 1 and 4 weeks post operation. The average peak systolic velocity (PSV) of the bilateral external iliac artery blood flow was 10 cm/s and 10.3 cm/s at 1 and 4 weeks post operation, respectively. Ultrasonography showed that the flow resistance indexes (RIs) on the right side and left side were 0.35 and 0.38 at 4 weeks post operation, respectively. The average uterine length was 2.8cm. Subsequently, ultrasonography showed that the graft uterus in the animal had normal gross morphology. The menstruation resumed normally within 2 months after surgery, and two of the animals had regular menstruation periods. Premenstrual ultrasound revealed endometrial edema in the rhesus monkey.

### Histological by biopsy

**Fig 5:**
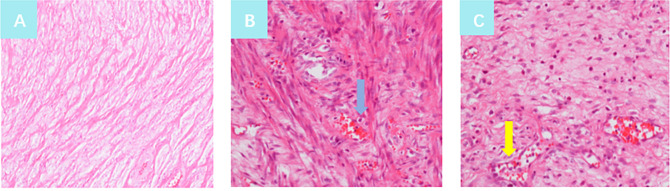
Histopathological findings in biopsy specimens of the cervix before surgery, 5 days and 25 days after surgery. A: Normal cervix, showing no marked inflammatory cell infiltration (original magnification ×20); B: Histopathological findings in a biopsy specimen of the cervix at 5 days after surgery, showing inflammatory findings consisting of lymphocytes and fresh bleeding (blue arrow, original magnification ×20); C: Histopathological findings in the biopsy specimen of the cervix at 25 days after surgery, showing inflammatory findings with lymphocytes and remote hemorrhage (yellow arrow, original magnification ×20).

Histopathologically, there was no abnormality in the cervix (Fig 5). Inflammatory findings with lymphocytes and fresh or remote hemorrhage could be found in the biopsy specimen of the cervix after surgery. This was a normal postoperative inflammatory response.

## Discussion

UTx has been performed in humans in several countries. However, this surgery is still considered to be at the experimental stage, since several clinical and technical issues remain to be resolved [14]. In this context, several aspects involved in UTx should be thoroughly addressed in animal models [15]. Over the last few decades, experimental UTx has been performed in rodents, rabbits, and large domestic animals such as sheep and pigs. Notably, pregnancies and births have been achieved in some of these cases, including in a mouse model in a syngeneic setting [16]. Since nonhuman primates are similar to humans in terms of anatomy and physiology, experiments in nonhuman primates are particularly appropriate for evaluated UTx in terms of clinical applications. However, there are some limitations in the experimental use of nonhuman primates for UTx [10, 11]. Female rhesus monkeys are smaller than humans; thus, complex vascular dissection of the pelvic floor is necessary for UTx. Furthermore, sophisticated surgical techniques are required in a rhesus model. In this study, the operation times of rhesus monkeys No. 3 and No. 4 were significantly shorter than those of rhesus monkeys No. 1 and No. 2. This difference can be ascribed to the accumulative experiences on the basis of initial practice, namely, that the surgeons had become more familiar with the anatomy of the pelvis and uterus-related anatomy of the rhesus monkey. With the continuously increasing proficiency of vascular anastomosis surgery, the rhesus UTx could be shorter and less deleterious to the animal. These improvements are reflected in the course of this study.

Some difficulties are also encountered in the postoperative management of this study. Immunosuppressive agents may cause unwanted side effects to blood cells, which would subsequently affect graft survival. However, it is difficult to orally administer these agents to nonhuman primates as scheduled. Oral delivery via enteral feeding is problematic because of the serious anorexia that occurs after invasive surgery and the side effects of the agents. Intravenous administration is a simple method in humans, but this would be difficult to control in animal models without anesthesia. Moreover, postoperative examinations, including echography and blood tests, cannot be regularly performed because sedation is frequently required. In this study, we mixed these immunosuppressants with food so that they could be ingested and absorbed by the animal. However, two to three days post operation, the animal had a poor appetite, which could explain the reduced absorption of immunosuppressive drugs. In 2002, cyclosporin maintenance was used in the first case of UTx in a human patient [17]. Most of the subsequent studies of human UTx have used tacrolimus in combination with mycophenolate mofetil. One previous study also used everolimus to reduce the dose of tacrolimus being administered [18]. In human UTx studies, steroids are usually commenced and weaned and reintroduced in cases complicated by rejection [19].

In this study, dexamethasone at a dose of 5 mg/day was administered orally to all animals on the day before surgery. The dexamethasone treatment lasted for two weeks post operation. MMF, CyA, and tacrolimus were also administered orally to prevent potential rejection in these recipients. Most UTx surgeries performed so far are based on the laparotomy approach [20]. These minimally invasive surgical techniques, including robotic and laparoscopic techniques, have been used in living UTx donor surgery [21, 22]. In human UTx donor surgery, the graft arteries are the uterine artery and adjacent internal iliac artery. Since the uterine artery is just 25 mm, the first case of UTx used the saphenous vein with a length of 60 to 80 mm to extend the uterine artery [17]. In this study, the graft arterials of three rhesus monkeys were the uterine artery and adjacent internal iliac artery. The graft arterial in one rhesus (No. 2) was separated to the level of the right common iliac artery. The donor animal (No. 2) had lameness and was unable to climb until one month after surgery. However, no necrosis was observed in the right lower limb. This rhesus gradually regained normal walking and climbing abilities one month after the operation. The early human UTx surgical technique separated the uterine veins to the level of the anterior division of the internal iliac vein [23]. This strategy of donor surgery for graft veins costs more in terms of operation time and carries a high risk of venous thrombosis [23].

In recent human UTx studies, several modifications have been made to improve venous drainage using ovarian/utero-ovarian veins. This surgical strategy for graft veins requires a shorter operation time since complex dissection around the uterine venous plexus and ureter is avoided [18, 24, 25]. In our study, the graft veins were uterine veins in animals No. 1 and No. 2, and ovarian/utero-ovarian veins in animals No. 3 and No. 4. The operation times for animals No. 3 and No. 4 (210 min and 220 min, respectively) were shorter than those of animals No. 1 and No. 2 (260 min and 250 min, respectively). These findings are consistent with previous reports. In human UTx, if the donor is younger, the removal of longer uterine and ovarian veins may affect ovarian function. In our study, we also found that the retained uterine and ovarian meridians had larger diameters and were more suitable for lavage. These uteri became pallor more rapidly.

The present study is the first report of allotransplant UTx performed in a rhesus model. We successfully developed a stable transplantation technique by performing vascular end-to-side anastomosis with internal and external iliac arteries. In this study, all donors and recipients were safe during transplantation surgery. The transplanted uterus had normal blood flow as evaluated by macroscopic appearance during surgery and ultrasonography after surgery. Another advantage of this study is that the pelvic vessels of the rhesus monkey are similar to those of humans. Although the rhesus model may not correspond directly to humans, the rhesus model is still important for addressing clinical issues regarding human UTx. These experimental data contribute to further successful clinical utilization of UTx.

## Conclusion

This report shows the safety and feasibility of allogeneic UTx technology in the rhesus model. No organ or vessel injury was observed in any of the donors or recipients. This is the first report to evaluate the functional recovery of a rhesus model after allogeneic UTx. We hope this research will provide a scientific foundation for future studies in primates and provide preclinical insights for human UTx.

## Supporting information

S1 Fig The transplanted uterus was evaluated by transabdominal ultrasonography one week after operation. (TIF)

S2 Fig The blood flow of the transplanted uterus was evaluated by transabdominal ultrasonography one week after operation. (TIF)

S3 Fig The transplanted uterus was evaluated by transabdominal ultrasonography four weeks after operation. (TIF)

S4 Fig The blood flow of the transplanted uterus was evaluated by transabdominal ultrasonography four weeks after operation. (TIF)

S1 Table Uterus transplantation surgery time in 4 rhesus monkeys. (DOCX)

S2 Table Uterine length of 4 rhesus monkeys. (DOCX)

S3 Table PSV on the bilateral external iliac artery of 4 rhesus monkeys. (DOCX)

S4 Table EDV on the bilateral external iliac artery of 4 rhesus monkeys. (DOCX)

S5 Table RIs on the bilateral external iliac artery of 4 rhesus monkeys. (DOCX)
