# Supplementary figures and images for: Allogeneic uterus transplantation in a rhesus model: A short-term graft viability study
Source: PLoS One. 2020 Dec 17;15(12):e0243140. doi: 10.1371/journal.pone.0243140 (PMC7746281; doi:10.1371/journal.pone.0243140)

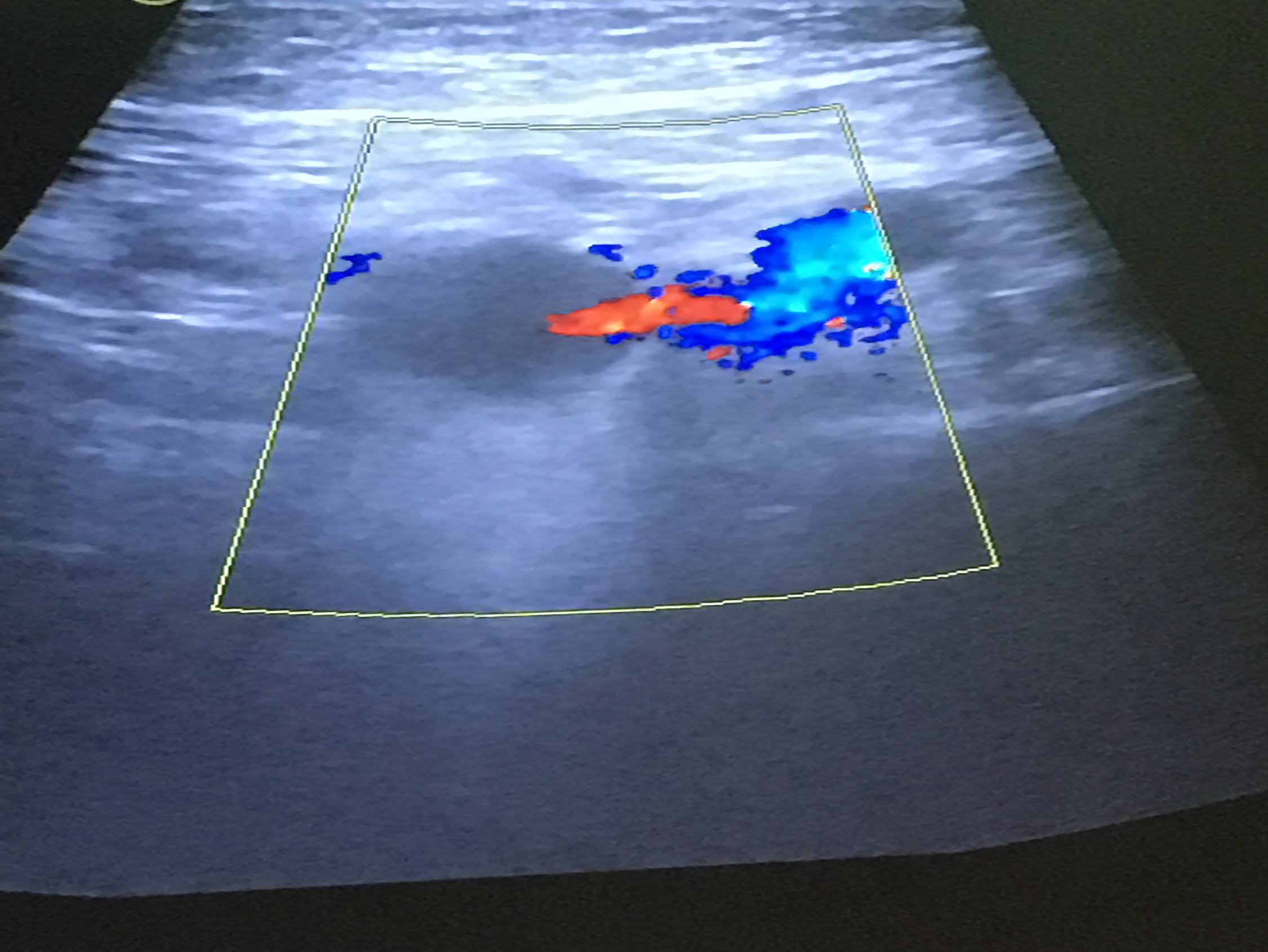

Supplement: S1 Fig — (TIF) [file pone.0243140.s001.tif]

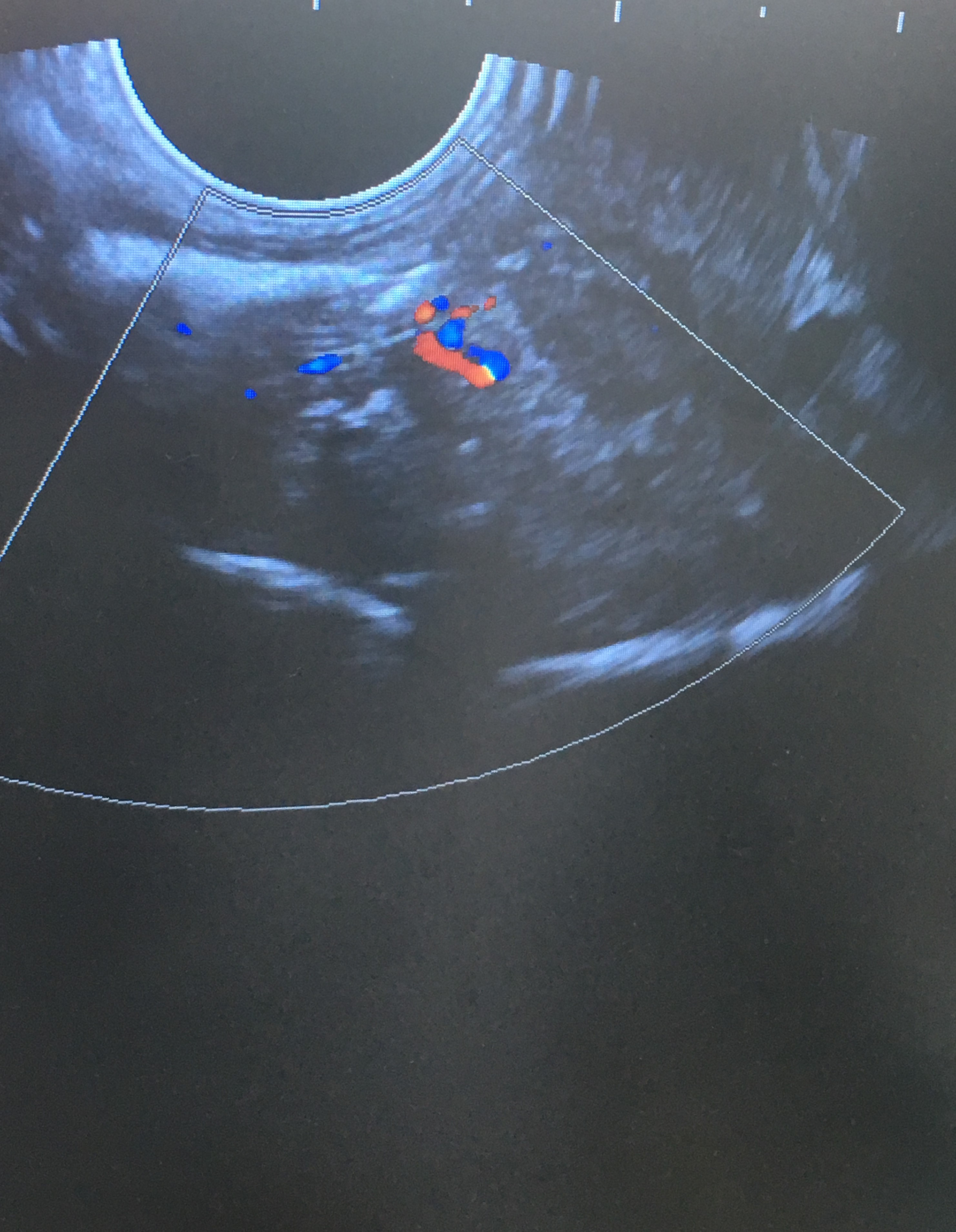

Supplement: S2 Fig — (TIF) [file pone.0243140.s002.tif]

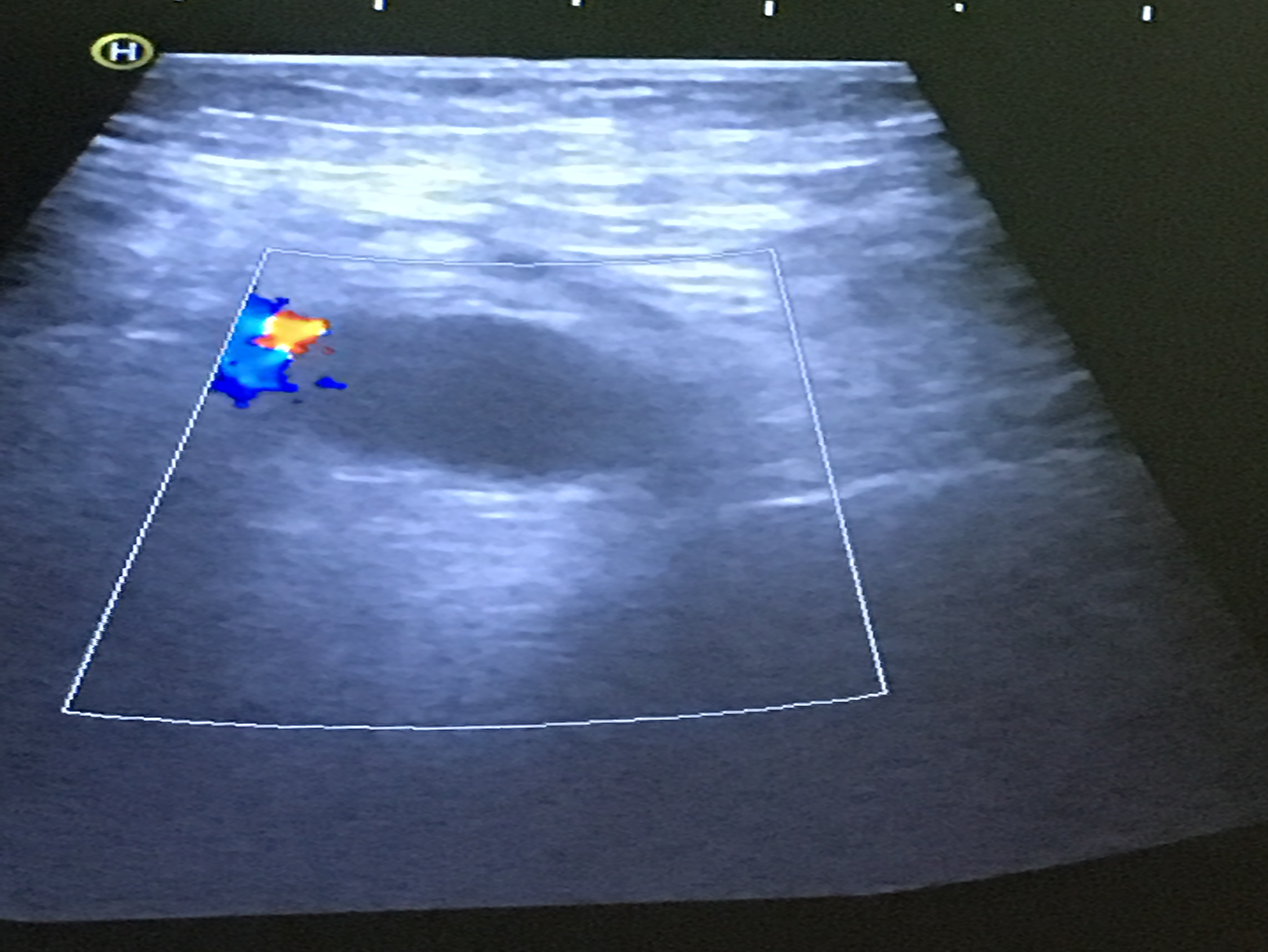

Supplement: S3 Fig — (TIF) [file pone.0243140.s003.tif]

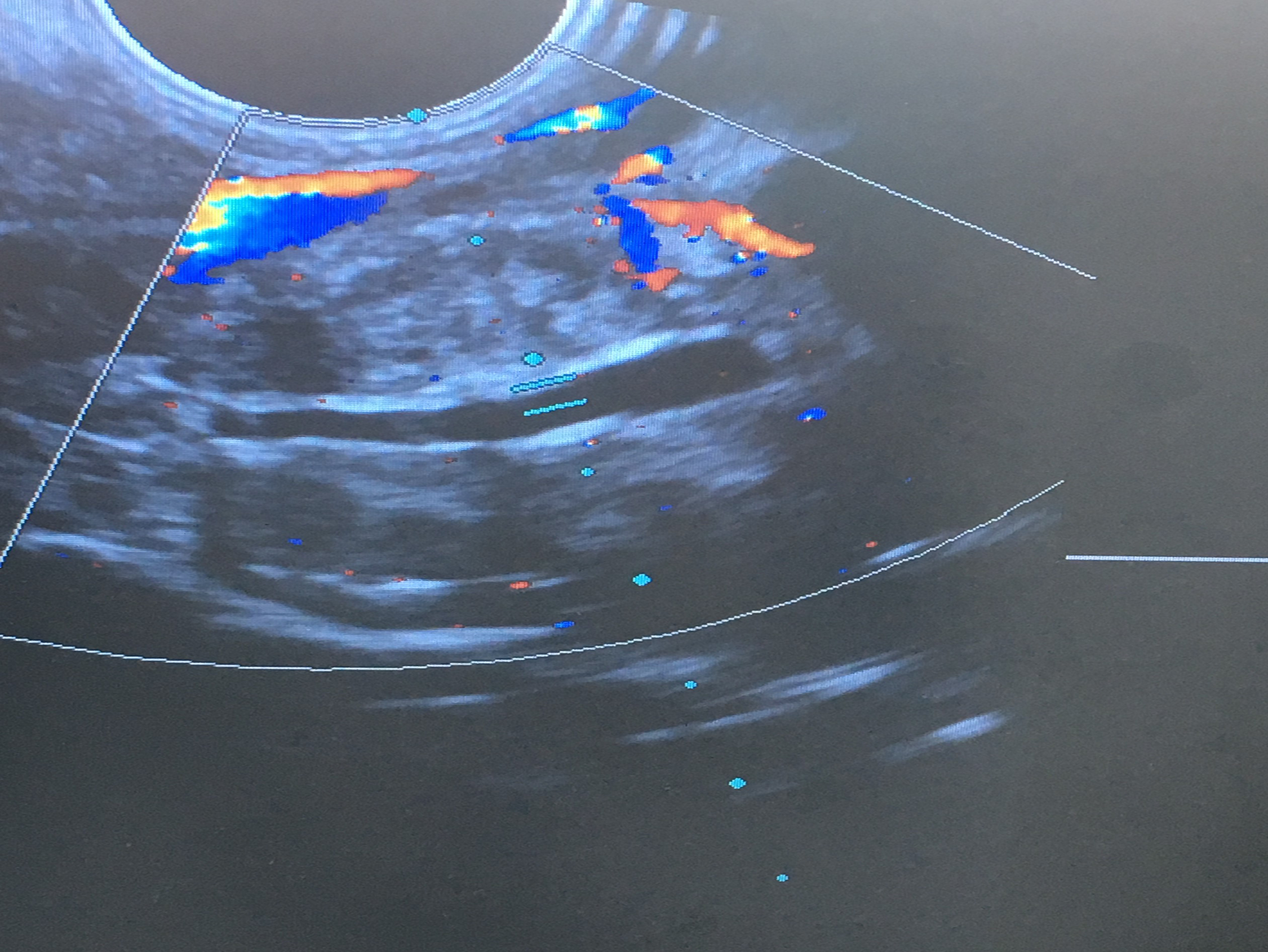

Supplement: S4 Fig — (TIF) [file pone.0243140.s004.tif]
