# Supplementary material for: Allogeneic uterus transplantation in a rhesus model: A short-term graft viability study
Source: PLoS One. 2020 Dec 17;15(12):e0243140. doi: 10.1371/journal.pone.0243140 (PMC7746281; doi:10.1371/journal.pone.0243140)
Supplement: S1 Table — (DOCX) [file pone.0243140.s005.docx]

**S1 Table. Uterus transplantation surgery time in 4 rhesus monkeys.**

warm ischemia 1 (donor): ischemia during implantation, from removal of the organ from ice until reperfusion; warm ischemia 2 (recipient): from cold storage to transplant vascular anastomosis completion and re-warming; cold ischemia: from organ retrieval to before vascular anastomosis.

|  | 1 | 2 | 3 | 4 |
| --- | --- | --- | --- | --- |
| **The start time of donor surgery** | 8:30 | 8:30 | 8:30 | 8:30 |
| **The start time of retrieval donor surgery** | 9:45 | 9:38 | 9:33 | 9:44 |
| **The end time of retrieval donor surgery** | 12:17 | 12:08 | 11:28 | 11:39 |
| **The end time of donor surgery** | 12:50 | 12:40 | 12:00 | 12:10 |
| **The start time of cold ischemia** | 12:27 | 12:19 | 11:35 | 11:47 |
| **The end time of cold ischemia**  **/ The start time of vascular anastomosis** | 14:38 | 14:19 | 12:10 | 12:47 |
| **Vascular anastomosis rewarming** | 16:31 | 16:22 | 13:32 | 14:16 |
| **The end time of vascular anastomosis** | 17:10 | 16:59 | 14:05 | 14:42 |
| **The end time of recipient surgery** | 17:30 | 17:21 | 14:32 | 15:04 |
| **Hemorrhage** | 20ml | 30ml | 15ml | 15ml |
