# Supplementary material for: Allogeneic uterus transplantation in a rhesus model: A short-term graft viability study
Source: PLoS One. 2020 Dec 17;15(12):e0243140. doi: 10.1371/journal.pone.0243140 (PMC7746281; doi:10.1371/journal.pone.0243140)
Supplement: S2 Table — (DOCX) [file pone.0243140.s006.docx]

**S2 Table. Uterine length of 4 rhesus monkeys**

| NO. | uterine length | |
| --- | --- | --- |
|  | 1 week post operation | 4 weeks post operation |
| 1 | 2.9cm | 2.9cm |
| 2 | 2.9cm | 2.9cm |
| 3 | 2.8 cm | 2.8 cm |
| 4 | 2.6 cm | 2.6 cm |
| average | 2.8 cm | |
