# Supplementary material for: Allogeneic uterus transplantation in a rhesus model: A short-term graft viability study
Source: PLoS One. 2020 Dec 17;15(12):e0243140. doi: 10.1371/journal.pone.0243140 (PMC7746281; doi:10.1371/journal.pone.0243140)
Supplement: S3 Table — (DOCX) [file pone.0243140.s007.docx]

**S3 Table. PSV on the bilateral external iliac artery of 4 rhesus monkeys**

| NO. | 1 week post operation | | 4 weeks post operation | |
| --- | --- | --- | --- | --- |
|  | PSV on the right external iliac artery | PSV on the left external iliac artery | PSV on the right external iliac artery | PSV on the left external iliac artery |
| 1 | 10.2cm/s | 9.8cm/s | 10.3cm/s | 10.1cm/s |
| 2 | 10.1cm/s | 10cm/s | 10.2cm/s | 10.2cm/s |
| 3 | 10.1cm/s | 9.7cm/s | 10.7cm/s | 10.4cm/s |
| 4 | 9.9cm/s | 10.2 cm/s | 10 cm/s | 10.5 cm/s |
| average | 10 cm/s | | 10.3 cm/s | |

PSV: peak systolic velocity
