# Supplementary material for: Allogeneic uterus transplantation in a rhesus model: A short-term graft viability study
Source: PLoS One. 2020 Dec 17;15(12):e0243140. doi: 10.1371/journal.pone.0243140 (PMC7746281; doi:10.1371/journal.pone.0243140)
Supplement: S4 Table — (DOCX) [file pone.0243140.s008.docx]

**S4 Table. EDV on the bilateral external iliac artery of 4 rhesus monkeys**

| NO. | 1 week post operation | | 4 weeks post operation | |
| --- | --- | --- | --- | --- |
|  | EDV on the right external iliac artery | EDV on the left external iliac artery | EDV on the right external iliac artery | EDV on the left external iliac artery |
| 1 | 6.5cm/s | 6.1cm/s | 6.6cm/s | 6.4cm/s |
| 2 | 6.7cm/s | 6.2cm/s | 6.7cm/s | 6.3cm/s |
| 3 | 6.7cm/s | 6.2cm/s | 6.7cm/s | 6.4cm/s |
| 4 | 6.7cm/s | 6.1 cm/s | 6.7 cm/s | 6.4cm/s |

EDV: end diastolic velocity
