# Supplementary material for: Allogeneic uterus transplantation in a rhesus model: A short-term graft viability study
Source: PLoS One. 2020 Dec 17;15(12):e0243140. doi: 10.1371/journal.pone.0243140 (PMC7746281; doi:10.1371/journal.pone.0243140)
Supplement: S5 Table — (DOCX) [file pone.0243140.s009.docx]

**S5 Table. RIs on the bilateral external iliac artery of 4 rhesus monkeys**

| NO. | 1 week post operation | | 4 weeks post operation | |
| --- | --- | --- | --- | --- |
|  | RIs on the right external iliac artery | RIs on the left external iliac artery | RIs on the right external iliac artery | RIs on the left external iliac artery |
| 1 | 0.36 | 0.38 | 0.36 | 0.37 |
| 2 | 0.34 | 0.38 | 0.34 | 0.38 |
| 3 | 0.34 | 0.36 | 0.37 | 0.38 |
| 4 | 0.32 | 0.40 | 0.33 | 0.39 |
| average | 0.34 | 0.38 | 0.35 | 0.38 |

RIs: flow resistance indexes

The formula: RIs=PSV-EDV**/**PSV
